# Antibacterial Effects of Erbium Chromium Laser along with/without Silver Nanoparticles in Root Canals Infected by *Enterococcus faecalis*

**DOI:** 10.1155/2021/6659146

**Published:** 2021-04-07

**Authors:** Seyedeh Sareh Hendi, Negin Amiri, Banafsheh Poormoradi, Mohammad Yousef Alikhani, Saeid Afshar, Maryam Farhadian

**Affiliations:** ^1^Department of Endodontics, Dental School, Hamadan University of Medical Science, Hamadan, Iran; ^2^Hamadan University of Medical Sciences, Hamadan, Iran; ^3^Dental Research Center, Department of Periodontology, Dentistry Faculty, Hamadan University of Medical Sciences, Hamadan, Iran; ^4^Microbiology Department, Faculty of Medicine, Hamadan University of Medical Science, Hamadan, Iran; ^5^Department of Molecular Medicine and Genetics, School of Advanced Technologies in Medicine, Molecular Medicine Research Center, Hamadan University of Medical Sciences, Hamadan, Iran; ^6^Department of Biostatics, School of Public Health and Research Center for Health Sciences, Hamadan University of Medical Science, Hamadan, Iran

## Abstract

This study investigates the antibacterial effects of erbium chromium laser at 2780 nm, silver nanoparticles, and erbium chromium along with silver nanoparticles on *Enterococcus faecalis* in comparison with sodium hypochlorite. In the present study, 90 extracted human single-rooted teeth were selected and standardized to a length of 15 mm. The canals were prepared by V-taper Gold rotary files and then incubated with *E. faecalis* for 21 days. The samples were divided into four experimental groups including hypochlorite sodium, silver nanoparticle, erbium chromium laser, and erbium chromium laser along with silver nanoparticle groups. Results showed that there was a significant reduction in colony count for all groups after interventions. Moreover, there was a significant reduction in the colony count for sodium hypochlorite group in comparison with another groups, and this group showed the highest reduction of colony count. There was a significant difference between silver nanoparticles and erbium chromium laser groups in colony count. According to the results, the silver nanoparticles offered strong antibacterial effects on E. *faecalis* and therefore can decrease bacterial colonies, while the use of the laser, despite the reduction of the bacterial colony, could not be sufficiently used for disinfection of root canal system.

## 1. Introduction

*Enterococcus faecalis* (*E. faecalis*) is a resistant bacterium that is able to live under various nutritional conditions. This bacterium has a high resistance to cleaning agents of root canal [1]. The main purpose of root canal treatment is to disinfect root canals and the three-dimensional structure of dentinal tubules [2–4]. Given the disadvantages of conventional root canal treatments, new methods such as lasers have received much attention from scientists in recent years. Multiple laser systems can be used as auxiliary tools to promote root canal disinfection. In addition, laser beams can penetrate deeply into the dentinal tubules without being absorbed by the hard tooth tissue, leading to a reduction in the bacterial population by 63% at a depth of 750 *μ*m [5, 6]. The American Food and Drug Administration (FDA) has recommended the use of erbium chromium laser for cleaning and shaping the root canal space. The laser can remove hard calcified tissue by emitting infrared beams at a wavelength of 2780 nm in combination with water spray. The erbium chromium laser absorbs a lot of water and can adsorb the water inside and around tissues. Therefore, the Er, Cr : YSGG laser can be more effective in the presence of water [7, 8]. Sodium hypochlorite is the most important material used in teeth root canal cleaning. Although it has many advantages, such as broad bactericidal spectrum and high ability for removing organic debris, but its antibacterial activity can be inactivated by the dentin or biological masses existing in the root canal, it needs to be frequently replaced and renewed. Moreover, root canal cleaning with sodium hypochlorite reduces the bond strength between dentin and resin [9, 10].

Silver nanoparticles have various medical applications due to their antibacterial and anti-inflammatory properties. The size of these particles ranges from 10 to 100 nm and they have antibacterial effects against Gram-negative and positive bacteria and also multidrug-resistant bacteria [10]. The mechanism of action of these particles is attributed to the release of silver ions, which are highly reactive and can react with the cell wall. The silver ions can form structural changes in the bacterium through the binding of these ions to tissue proteins [10, 11]. Therefore, the use of silver nanoparticles has more antimicrobial effects over common medicines and disinfectants [12].

The root canal diseases are associated with pain, inflammation, and discomfort of the patient. On the other hand, *E. faecalis* is a resistant bacterium that causes recurrence and root canal infection. Therefore, the present study was aimed at introducing an efficient method to remove and eradicate this bacterium from root canal. In recent years, the effective role of silver nanoparticles on bacterial destruction and root canal cleaning has been investigated. Moreover, recent advances in the use of laser in dentistry have received much interest in the last couple of decades. In this regard, in the present study, we aimed to evaluate the antibacterial effects of silver nanoparticles along with erbium chromium laser at 2780 nm on *E. faecalis* existing in root canal.

## 2. Materials and Methods

### 2.1. Materials

The following materials were used: diamond disk (DFS Diamon Co., Germany) for removing the crown, K-files (Mani Inc., Japan), and rotary files (V-Taper Gold, Shanghai Fanta Dental, China) for preparation of the root canals. Normal saline (sodium chloride, 0.9%) (Shahid ghazi Co., Tabriz, Iran), EDTA 17% (Maraboon, Iran), and 5% sodium hypochlorite (Paxan Co., Tehran, Iran) for irrigation of root canals. Eppendorf tube (Amin Co., Iran) and Acryle (Acropars Co., Iran) for fixing the roots. Standard strain of *E. faecalis* (ATCC29212), Trypticase soy broth; TSB (Hi-Media Laboratories, Mumbai, India), Tween 80 solution (Samchun Pure Chemical, Korea), PBS (Phosphate-buffered saline), and sheep blood agar plates (Hi-Media Laboratories, Mumbai, India) for microbiologic experiments. Sodium thiosulfate solution (Samchun Pure Chemical, Korea) for neutralizing sodium hypochlorite solution. ZetaSizer Nano ZS (ZEN3600) (Malvern, United Kingdom) for measuring Zeta potential. Erbium chromium laser (Biolase, Waterlase, USA).

### 2.2. Teeth Collection: Inclusion and Exclusion Criteria

The study was performed on 90 newly extracted single-canal human teeth and their single-canal was confirmed by radiography. The teeth were collected from comprehensive health centers. Sampling of single-canal anterior teeth was performed randomly based on the following inclusion criteria: teeth without any developmental defects, caries, and cracks or fractures. Moreover, nonaged teeth were selected to ensure that there is no dentin sclerosis and the calcified dental canal in the samples. Dental radiographs were taken to ensure that the selected teeth are single channel before operation, and teeth with curved roots were excluded.

### 2.3. Teeth Preparation

The samples were classified randomly into different treatment groups. In order to prepare the samples, their roots were cut at approximately 15 mm of apex using a diamond disk, and its working length was adjusted at 1 mm shorter than the apical foramen (14 mm). The root canals were first prepared up to No. 20 using manual K-files (Mani Inc, Japan), and 5% sodium hypochlorite (Paxan Co., Tehran, Iran) then prepared by rotary files (V-Taper Gold, Shanghai Fanta Dental, China) to obtain the original file. During the use of rotary files, the canals were washed with normal saline (sodium chloride, 0.9%) and 5% sodium hypochlorite. After preparation with files, the canals were washed with 1 mL of17% EDTA (Maraboon, Iran), 5 mL of normal saline, and 1 mL of 5% sodium hypochlorite and kept for 5 min in order to remove smear layer. Eventually, all canals were washed with 5 mL of normal saline. Afterwards, the roots of the teeth were fixed into a 2 mL microtube with acrylic (Acropars Co., Iran) and incubated at 121°C for 15 minutes [1].

### 2.4. Synthesis of AgPPENPs

To prepare silver nanoparticle suspension, pomegranate skin was dried in an oven at 50°C for 2 days, and to prepare the hydroalcoholic extract, 50 g of the pomegranate skin was soaked in 500 mL of alcohol (70%) at room temperature for 72 hours. The extract of pomegranate powder was then extracted using Whitman No. 40 filter papers. In order to separate the fine suspended particles from the extract, a centrifuge was used. A certain amount of silver nitrate and the extract were dissolved in 200 mL of water to prepare the desired concentrations (100 and 200 ppm). The color of the solution changed from colorless to gray, which indicated the successful synthesis of AgPPENPs [13].

### 2.5. Cell Toxicity Assay of PPE and AgPPENPs

To examine potential toxicity of formulation, RMPI medium in 5% CO_2_ of atmosphere with the temperature of 37°C was used and fibroblast cell line L929 was cultured within. The cultured cells were exposed to different concentration of PPE and AgPPENPs (100, 200, 300, 400, and 500 *μ*g/mL). We used MTT assay to evaluate viability of the cells. The cells were cultured in 96-well plates and incubated for 3 hours with MTT solution. To solubilize formazan particles, DMSO solution was also added to each well and absorption rate was read using ELISA reader in 580 nm.

### 2.6. Bacterial Inoculation of Root Canals

The standard strain of *E*. *faecalis* (ATCC29212) was cultured in 5 mL of Trypticase soy broth, TSB (Hi-Media Laboratories, Mumbai, India) and incubated at 37°C for 24 hours and a concentration equivalent of 0.5 McFarland standard from E. *faecalis* (1.5 × 108 CFU/mL) was provided. Then, 100 *µ*L of sterile TSB was added to the microtubule containing sterile tooth and 150 to 200 *µ*L of bacteria suspension (1.5 × 108 CFU/mL) was added to microtubes containing teeth. 30 *µ*L of the culture containing about 10^9^*E. faecalis* bacteria was added to the canals. The samples were then incubated at 37°C for 21 days to form biofilm. Every two days, 30 *µ*L of TSB suspension containing *E*. *faecalis* was removed from the canal and replaced with fresh sterile TSB under sterile conditions [8, 14]. Congo red coloring was used for confirming biofilm formation.

### 2.7. Experimental Procedures

Group 1: cleaning with 5% sodium hypochlorite: in this group, the root canal of the teeth was cleaned routinely with injecting 5 mL of 5% sodium hypochlorite solution (Snow, Paxan Co., Tehran, Iran) in apical third of canal (one or two mm from the foramen) using a 30-G insulin syringe and kept in the canal for 5 min. Then, 1 mL of 5% sodium thiosulfate solution (Samchun Pure Chemical, Korea) was injected into neutralize sodium hypochlorite solution and kept in the canal.

Group 2: cleaning with silver nanoparticles: the root canals were washed with 5 mL of the prepared silver nanoparticle suspension with a concentration of 100 ppm (with mean particle size of 20 nm). For this reason, an insulin syringe (Supa, Iran) was utilized and kept in the canal for 5 min.

Group 3: using 2780 nm erbium chromium laser: the root canals of the teeth were cleaned using erbium chromium laser (Biolase, Waterlase, USA) at a wavelength of 2780 nm, 0.0% of water, 15% of air, and 75 watts of power. The power density was set at 2.5 watt/cm^2^ and energy density was equal to 25 J/cm^2^. The laser head with a diameter of 200 *μ*m and 1 mm shorter than the apex (Biolase, Waterlase, USA, RFT2/Hmode) was used continuously with rotational and epicocoronal motions for 30 sec. This process was performed three times with an interval of 15 sec between each laser irradiation.

Group 4: simultaneous use of erbium chromium laser and silver nanoparticles: in this group, the root canals were cleaned with 5 mL of the synthetized silver nanoparticles at a concentration of 100 ppm (with mean particle size of 20 nm) using an insulin syringe (Supa, Iran). After 5 min, the erbium chromium laser with a wavelength of 2780 nm was applied for 30 sec, and this operation was conducted three times with an interval of 15 sec between each irradiation similar to the group.

Group 5: negative control group (normal saline): root canals were washed with 5 mL normal saline using insulin syringe.

### 2.8. Initial Sampling

To standardize the bacterial prototype after the extraction of TSB and washing the canal with 5 ml normal saline, root canals were filled with sterile saline solution using a 30-G syringe, and dentin was scraped from inside the canals using a #40 Hedstrom file (Mani Inc, Tochigi, Japan). A #40 sterile paper point (Gapadent Co, Hamburg, Germany) was placed inside the canals for 60 seconds and was then transferred into a sterile microtube containing 1 mL Tween 80 solution (Samchun Pure Chemical, Korea) and vortexed for 20 seconds to isolate the bacterial biofilm. One hundred dilution was made from the first sample and cultured on blood agar medium and the plates were incubated at 37°C for 24 h and colony counts were reported according to CFU/mL (8).

### 2.9. Final Sampling

To standardize all groups, the canals were washed with 5 mL of normal saline and the solution was kept in the root canal for 30 sec. Then, the sampling was performed in accordance with the primary sampling method and colony counts were reported according to CFU/mL.

### 2.10. Statistical Analysis

Accordingly, SPSS software (version 21) was utilized to analyze the data. For this purpose, the central dispersion indices for the number of live bacterial colonies were calculated and reported before and after the intervention. The decrease in bacterial colonies after the intervention in different groups was compared by Kruskal–Wallis nonparametric test. Moreover, changes in bacterial colonies before and after the intervention were recorded using Wilcoxon signed-rank test. Given the fact that the data were not in line with the normal distribution and presence of dispersed data in the colonies, nonparametric analyses were performed in the study. Type I error rate was set at 0.05 (*α* = 0.05) [10].

## 3. Results

### 3.1. Properties of AgPPENPs

The mean of zeta potential for the nanoparticles at pH = 7 was −28 mV.

**Figure 1:**
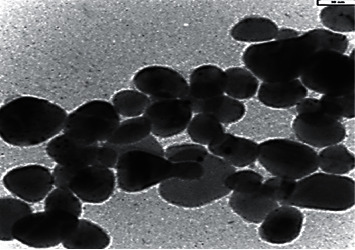
Transmission electron microscopy (TEM) image of the AgPPENPs (scale bar is 50 nm).

TEM image results showed that the mean diameter of NPs was 20 nm (Figure 1).

### 3.2. X-Ray Diffraction of AgPPENPs

**Figure 2:**
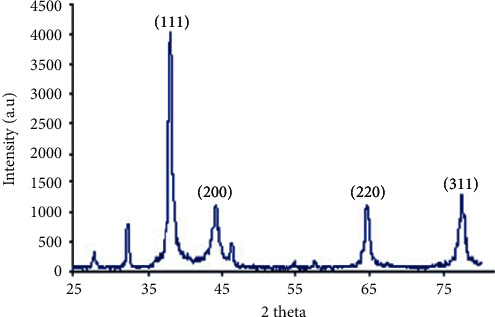
X-ray diffraction (XRD) pattern of AgPPENPs.

Figure 2 shows the XRD pattern of synthesized nanoparticles (AgPPENPs). Main peaks recorded at 2*θ* from 32.28, 38.18, 44.53, 64.68, and 77.58 are indexed as diffraction (111), (200), (220), and (311), respectively.

### 3.3. Cell Toxicity

**Figure 3:**
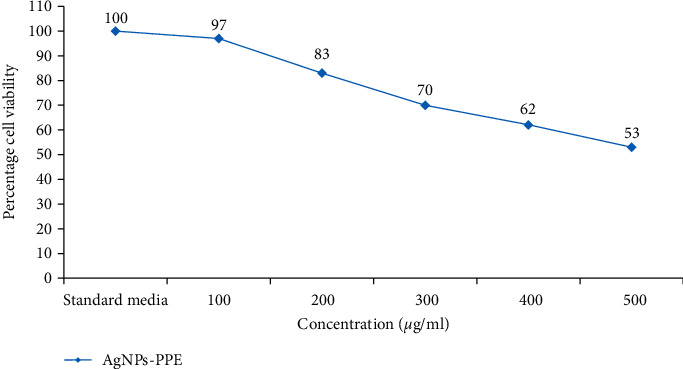
Cells viability of treatments with different concentrations of AgNPs-PPE and PPE after 48 hrs.

To assess the potential toxicity of synthesized nanoparticles, cell viability assay was applied. The results pointed that AgPPENPs could decrease cell viability in the concentration of 400 and 500 *μ*g/mL, whereas PPE could decrease toxicity of the particles lonely, where the particles became completely safe in the concentration of 400 *μ*g/mL without any remarkable toxicity (Figure 3).

### 3.4. Results

**Figure 4:**
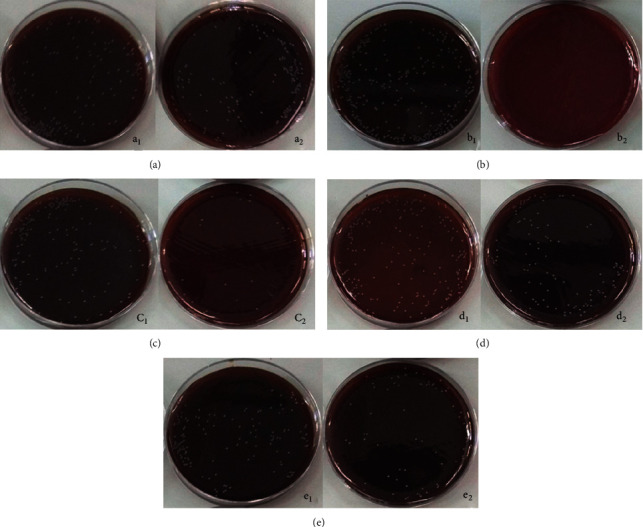
Bacterial culturing before and after intervention. (a) Negative control, a_1_ before a_2_ after. (b) Hypochlorite sodium, b_1_ before b_2_ after. (c) Nanosilvers, c_1_ before c_2_ after. (d) Erbium chromium laser, d_1_ before d_2_ after. (e) Nanosilvers and erbium chromium laser, e_1_ before e_2_ after.

According to the results of Wilcoxon test, there was a significant decrease in the number of bacterial colonies after the intervention (*p* < 0.05).  Table 1 and Figure 4 present descriptive values of *E. faecalis* colonies before and after the intervention and reduction percentage of colony count (RCC) in each studied group. The highest reduction percentage of E. *faecalis* colonies was observed in the sodium hypochlorite group, indicating that this disinfectant acted as the best disinfectant with an efficiency of 100%, and the lowest decrease was observed in the erbium chromium laser group with an efficiency of 47.9%.

Kruskal–Wallis test was used to compare the performance of different interventions with respect to the reduction percentage of bacterial colonies based on the mean values of the reduction of colony count (RCC). The results of this test showed that there was a significant difference between the reductions of bacterial colonies between different groups (*p* = 0.03).

Based on this result, Mann–Whitney test was used to compare the two groups. In most cases, the type I error rate (*α*) is considered to be 0.05, but in the Mann–Whitney test by Bonferroni adjustment *α* was calculated according to the number of groups. In this study, according to the number of the groups (5 groups including four interventions and one control group), the value of *α* was calculated to be 0.005. Therefore, the *p* values less than 005 indicate a significant difference between the groups and the *p* values greater than 0.005 indicate no significant difference between the groups (Table 2).

Results of Mann–Whitney test with Bonferroni adjustment showed that there was a significant difference between the sodium hypochlorite and silver nanoparticle groups with negative control group (*p* < 0.005), while the difference between the erbium chromium laser and erbium chromium laser along with silver nanoparticle groups with negative control group was not significantly different (*p* > 0.005). Moreover, there was a significant difference between the effect of sodium hypochlorite on reduction of bacterial colonies and other groups (*p* < 0.001). The erbium chromium laser along with silver nanoparticle group had no significant difference with negative control group (*p* = 0.102) and erbium chromium laser group (*p* = 0.152). The erbium chromium laser group showed a lower effect compared with other groups. Also, there was no significant difference in the reduction of bacterial colonies between the silver nanoparticles group and silver nanoparticles along with erbium chromium laser group (*p* = 0.009).

## 4. Discussion

This study evaluated the antimicrobial effect of erbium chromium laser, silver nanoparticles, and their simultaneous applications on *E. faecalis* bacteria. Moreover, their effects were compared to sodium hypochlorite as the gold standard. The results showed that there was a significant decrease in the number of bacterial colonies between after and before using erbium chromium laser at 2780 nm (*p*_value_ < 0.001). But the reduction percentage of bacterial colonies in the erbium chromium laser group was less than the sodium hypochlorite group (RCC = 47.9). Also, the comparison of this group with the negative control group showed no significant difference (*p*_value_ = 0.943).

In Kasić et al.'s [14] study, the YSGG and Er : YAG lasers significantly reduced the number of microorganisms (*p* < 0.05). In the mentioned study, the high efficiency in the use of laser for cleaning root canal compared with the present study can be attributed to the rapid, turbulent, and mechanical movements of fluid inside the root canal, which facilitate the removal of the bacterial biofilm in root canal. The low efficiency of the erbium chromium laser in the present study can be related to the adsorption of laser power in the initial depth of dentin (at a depth of 400–300 *μ*m). Also, in the present study, the dental canal was dried prior to laser irradiation by paper No. 40, which can be considered as another probable reason for the low efficiency of laser application, because, in the mentioned study, during the irradiation of lasers of Er, Cr: YSGG and Er: YAG, 5 mL of saline was also injected by a 27-gauge needle attached to a syringe with intra-canal pressure. In this study, the dental canal was dried with absorbent paper points (No. 40).

Due to the high absorption of erbium laser radiations by water and hydroxyapatite, and thus the reduction of the laser thermal effect, lasers are more efficient in the dried canals. In general, lasers such as erbium chromium laser can kill bacteria through their thermal effects. Therefore, the moisture can act as a reducing agent and subsequently reduces the lethal effect of lasers on bacteria [15].

According to the results of Wanda Gordon et al.'s study, the use of Er, Cr: YSGG laser at 240 nm and an exposure time of 240 sec showed higher efficiency than sodium hypochlorite in dried root canal, which resulted in 99.7% reduction in bacterial count [7]. The difference between these results and the present study may be due to differences in the method used, because, in the mentioned study, dentin cylinders with a length and diameter of 5 mm were used, but in our study the root length was longer (15 mm) and radiation area was greater (14 mm). Laser light is emitted in a specific direction from the laser head or it is diverted from the straight direction by an angle of 18–20 degrees. The use of these inconsistent radiations makes it difficult to achieve a constant lighting condition in all dentinal surfaces of the root canal [16]. El-Gendy et al. reported that sodium hypochlorite had the highest bactericidal effect, which is consistent with our findings [17].

In Eldeniz et al.'s study, sodium hypochlorite was used in root canal for 15 min, while in the present study this detergent was kept in canal for 5 min. Given the toxic effects of sodium hypochlorite, it has been suggested to be used for short contact times in dental applications. In Eldeniz et al.'s [18] study, Er, Cr: YSGG laser showed much better efficiency than the control group (95% vs. 0%), while, in our research, the bactericidal efficiency of normal saline in the control group (49%) was higher than the erbium chromium in laser group at 2780 nm (47.9%) [19]. The better efficiency in the laser groups in Eldeniz et al.'s study may be due to the short inoculation time (48 h) in the root canal compared with 21 days in the present study, where the bacterial resistance to the cleaning agents increases with increasing the incubation time in the root canal and subsequently reduces the bactericidal effect of the used cleaning agents. In addition, in Eldeniz et al.'s study, the low efficiency in the control group can be attributed to receiving no treatment or intervention, whereas, in the present study, the control group received 5 mL of normal saline and washed for 5 min.

The antimicrobial property of sodium hypochlorite depends on the concentration of unconjugated hypochlorous acid in the solution. The hypochlorous acid interacts with sulfhydryl groups in bacterial enzymes. By preventing the activity of these essential enzymes, their basic metabolic reactions are disrupted and results in bacterial death [20]. Moreover, the results of Gutknecht et al.'s study are consistent with our findings. Similarly to the above mentioned study, we found that the single erbium chromium laser did not have a sufficient bactericidal activity [21].

In Charannya et al.'s study, the efficiency of silver nanoparticles in the mentioned study was lower than the present study, which could be attributed to the use of low concentrations [19, 22]. Also, the size of the nanoparticles in the mentioned study was much larger (700–300 nm) than that of the present study (20 nm). In nanoparticle technology, the smaller particle size leads to a higher specific surface area and hence increases its antibacterial effect [23]. We et al. also reported that the antibacterial efficacy of silver nanoparticles against E. *faecalis* biofilm depends on the used method with emphasis on concentration and contact time [24].

There was a contradiction regarding the use of silver nanoparticles in cleaning root canals between our findings and the results of We et al.'s study, because the results of Wu et al.'s study showed that sodium hypochlorite 2% and 98.2% reduce the number of *Enterococcus faecalis* bacterial colonies and silver nanoparticles solution 1% and 5.6% decreased the bacterial load. But, in the present study, silver nanoparticles acted as a good cleaner and destroyed 83.15% of *Enterococcus faecalis* bacterial colonies and the reason for this difference can be the period for which the time solution remains in the root canal mentioned in Wu et al.'s study as 2 minutes while in the present study the silver nanoparticle solution remained in the canal for 5 minutes. Another reason is the percentage of silver nanoparticles solution used with the percent of 1% and 100% (pure solution) in Wu et al.'s and the present study, respectively. Although dentin parts were larger in our study (14 mm roots versus dentin parts (4 mm long, 4 mm wide, and 1 mm high)), the success of our study was much greater, emphasizing the importance of nanoparticle concentration and time [23, 24], while Xie et al. reported that sodium hypochlorite showed the highest efficiency for cleaning root canals, which is in agreement with our findings [25].

## 5. Conclusion

The results implied that the application of erbium chromium reduced the number of bacterial colonies and there was a difference between before and after laser treatment. But they were not as effective as sodium hypochlorite and did not significantly reduce the number of *E. faecalis* bacteria in the root canals. Moreover, the results demonstrated that the use of single erbium chromium laser and single silver nanoparticle was not sufficient to remove the bacteria from root canal. The use of silver nanoparticle suspension at the desired concentration and appropriate contact time eliminated more bacterial colonies compared with the negative control group. According to the results, it can be concluded that the higher concentrations or high contact times of silver nanoparticles with root canal can be used to achieve better antibacterial effect for cleaning root canals.

## Figures and Tables

**Table 1:** Descriptive values of *E. faecalis* colonies before and after intervention and reduction percentage of bacterial colonies in each studied group.

Groups	Time	Minimum	Maximum	RCC %	*p* value
Sodium hypochlorite	Before	10^3^ × 12	10^3^ × 27	100	0.001>
	After	0	0		

Silver nanoparticle	Before	10^3^ × 5	10^3^ × 60	83.15	0.001>
	After	0	10^3^ × 8		

Erbium chromium laser	Before	10^3^ × 11	10^3^ × 36	47.90	0.001>
	After	10^3^ × 3	10^3^ × 30		

Silver nanoparticles and erbium chromium lasers	Before	10^3^ × 4	10^3^ × 49	64.72	0.001>
	After	0	10^3^ × 13		

Negative control (washing with normal saline)	Before	10^3^ × 3	10^3^ × 58	49	0.005>
	After	10^3^	10^3^ × 41		

^*∗*^Wilcoxon test for determining significant differences in bacterial count before and after intervention.

**Table 2:** Results of Mann–Whitney test with Bonferroni adjustment for comparing the two groups.

Groups	RCC mean
	*p*_value_
Sodium hypochlorite, negative control group	0.001
Erbium chromium laser, negative control group	0.943
Silver nanoparticles, negative control group	0.001
Erbium chromium laser and silver nanoparticles, negative control group	0.102
Silver nanoparticles, sodium hypochlorite	0.001
Erbium chromium laser, sodium hypochlorite	0.001
Erbium chromium laser and silver nanoparticles, sodium hypochlorite	0.001
Silver nanoparticles, erbium chromium laser	0.001
Silver nanoparticles, erbium chromium lasers and silver nanoparticles	0.009
Silver nanoparticles and erbium chromium laser, erbium chromium laser	0.152

## Data Availability

The data can be accessible to the interested researchers by the corresponding authors on reasonable request.
